# Supplementary material for: Magnitude of meconium stained amniotic fluid and associated factors among women who gave birth in North Shoa Zone hospitals, Amhara Region, Ethiopia 2022
Source: PLoS One. 2024 Feb 14;19(2):e0297654. doi: 10.1371/journal.pone.0297654 (PMC10866510; doi:10.1371/journal.pone.0297654)
Supplement: S1 File — (PDF) [file pone.0297654.s003.pdf]

አስራት ወልደየስ ጤና ሳይንስ ካምፓስ
የ ጥናትና ምርምር ግምገማ ቢሮ

Asrat Weldeyes health Science Campus
Institutional Review Board office

ቁጥር/ Ref. No: **IRB 56/ 09 / 2014 ዓ.ም**

ቀን / Date: - **06 / June / 2022**

Protocol number: IRB-053

To: Mitku Tefera

Subject: Ethical approval of your research protocol

The IRB of Asrat Weldeyes Health Science campus, Debre Berhan University has reviewed your research project entitled **Magnitude of meconium stained amniotic fluid and associated factors among women who gave birth in north shoa zone hospitals Amhara region Ethiopia 2022.** This is to notify that this research protocol as presented to the IRB meets the ethical and scientific standards outlined in the national and international guidelines. Hence, we are pleased to inform you that your protocol is ethically cleared.

We strongly recommend that any significant deviation from the methodological details indicated in the approved protocol must be communicated to the IRB before they are implemented.

Institution Review Board (IRB) approval: period from June 06/2022 to December /30/2022.

With regards!

እዮስያስ የሺዓለም አሰፋ
Eyosiyas Yeshialem Asefa

ተቋማዊ የጥናትና ምርምር ግምገማ
ቦርድ አስተባባሪ
Institutional Review Board Coordinator

✉ 445 መልስ ሲጽፉልን የኛን ደብዳቤ ቁጥር ይጥቀሱ ☎251-011-8697135/ +251-913-83-68-69
Email: dbu@ethionet.et/ eyosi143@gmail.com In replying, please quote our Ref. No Fax- 011-681-20-65
