# Supplementary material for: Magnitude of meconium stained amniotic fluid and associated factors among women who gave birth in North Shoa Zone hospitals, Amhara Region, Ethiopia 2022
Source: PLoS One. 2024 Feb 14;19(2):e0297654. doi: 10.1371/journal.pone.0297654 (PMC10866510; doi:10.1371/journal.pone.0297654)
Supplement: Attachment — Submitted filename: Comments to Authors.docx [file pone.0297654.s006.docx]

Comments to Authors

Abstract

1. **Background**: Line number 22 &23: Says “In Ethiopia meconium-stained amniotic fluid is **under investigated** specifically in North Shoa Zone Amhara region Ethiopia”. Do you think that researches for each problem should be done in each zone of the region to say “well investigated”? Why the research findings in other zones of the region like North Gondar, East Gojjam South Wollo…cannot be applicable for North Shoa?
2. **Methods**: Line 27 &28 : “Two stage cluster sampling was used to recruit study participants”. Is cluster sampling technique appropriate for your research? Why you prefer it over systematic random sampling technique? Would you mind attaching the schematic presentation of the sampling procedure? Why you use a design effect of 2?
3. **Conclusion**: Line 38: “The magnitude of meconium-stained amniotic fluid, which was **high**”. What is your ground/reference frame to say “high”? What is “low” to you? Your recommendation is not in line with your finding: Did your research finding showed a gap on “intrapartum service” and “a delay on detecting meconium-stained amniotic fluid “ in the study area?
4. **Introduction**: line number 45-53: More than 9 lines/ two paragraphs taken from one source without paraphrasing it which is not appropriate writing.
5. **Methods and materials:** line 80: Better to incorporate how many Hospitals are there in your study setting and number of deliveries in each Hospital monthly.
6. **Line 99:** I am not comfortable with design effect and cluster sampling technique. I need a strong justification with schematic presentation of sampling procedure.
7. **Line 125:** what were the adjustments and modifications made to the tool after your pre-test?
8. **Lines 137-145:** All your operational definitions citation
9. **Result:** Table 2: Have you ever drinking Alcohol? Yes (69), No (541) and Have you drinking Alcohol currently Yes ?(148), No ( 457). A paradox result,
10. Can we measure behavioural factors with simple yes/no questionnaire?
11. Table: Can it add a value to your result using “gravidity and parity” at the same time as different factors? Look at your result again “Gravidity 2-4=305 and parity 2-4=358, how it could be? Is parity greater than gravidity?
12. Discussion: The literatures compared to your result should be written from local to global unlike that of literature writing style.
