# Supplementary material for: Magnitude of meconium stained amniotic fluid and associated factors among women who gave birth in North Shoa Zone hospitals, Amhara Region, Ethiopia 2022
Source: PLoS One. 2024 Feb 14;19(2):e0297654. doi: 10.1371/journal.pone.0297654 (PMC10866510; doi:10.1371/journal.pone.0297654)
Supplement: Attachment — Submitted filename: Response to Reviewers.docx [file pone.0297654.s007.docx]

**Letter of response**

**To all honourable reviewers and editors**

Dear Reviewers and Editors, We are very grateful for your relevant comments, questions and suggestions. Following this, we have provided our responses point by point as presented below.

**Authors Responses**

**1. Is the manuscripts technically sound, and do the data support the conclusions?

The manuscript must describe a technically sound piece of scientific research with data that supports the conclusions. Experiments must have been conducted rigorously, with appropriate controls, replication, and sample sizes. The conclusions must be drawn appropriately based on the data presented.**

**Reviewer #1: No**

**Reviewer #2: Yes**

**Authors Responses**

We rewrite the conclusion in such a way that it is drawn from the presented data.

2. Has the statistical analysis been performed appropriately and rigorously?

Reviewer #1: No

Reviewer #2: Yes

**Authors Responses**

We recalibrated our data analysis.

3. Have the authors made all data underlying the findings in their manuscript fully available?

The [PLOS Data policy](http://www.plosone.org/static/policies.action#sharing) requires authors to make all data underlying the findings described in their manuscript fully available without restriction, with rare exception (please refer to the Data Availability Statement in the manuscript PDF file). The data should be provided as part of the manuscript or its supporting information, or deposited to a public repository. For example, in addition to summary statistics, the data points behind means, medians and variance measures should be available. If there are restrictions on publicly sharing data—e.g. participant privacy or use of data from a third party—those must be specified.

Reviewer #1: Yes

Reviewer #2: Yes

4. Is the manuscript presented in an intelligible fashion and written in standard English?

PLOS ONE does not copyedit accepted manuscripts, so the language in submitted articles must be clear, correct, and unambiguous. Any typographical or grammatical errors should be corrected at revision, so please note any specific errors here.

Reviewer #1: No

Reviewer #2: No

**Authors Responses**

# We have tried to revise language editing through the document and it will further be edited in the process of write up for publication.

# Response to Reviewer #1

**1. Most of your study results have a wide range of AOR . Why it is happen, and also what is the implication in conclusion and recommendation in scientific world?**

Answer

This is an interesting question. As we know, the larger the odds ratio, the more likely the event is to be found with exposure. According to this, there are those who show a large AOR number in our study, for example, having tachycardia 6.2 times more likely to cause MSAF than normal and having bradycardia 3.1 times more likely to cause MSAF than a normal fetal heart rate. Based on this, we conclude that mothers with a baby's heart rate indicating tachycardia or bradycardia are vulnerable to MSAF, and we have recommended that health professionals pay extra attention to MSAF if there is such a labor complication during delivery.

**2. What makes different this paper other than the study design? Since there are a number of study conducted in Ethiopia.**

Answer

Respected, you are right,

- Many studies have been conducted in Ethiopia by cross-sectional and some case control, but this is different from the ones studied in the past.
- Previous studies only studied retrospective data, but our study used both primary and secondary data.
- We tried to include the variable that the previous studies did not include.
- We studied a large study area using large sample sizes. Because of these differences, the results of our study both in magnitude and associated factors are different from previous studies.

**3. What talents or techniques you used to improve the quality of paper during data collection, data enter and analysis.**

Answer

Thank you very much for your interesting question.

- Before doing the main data collection, we used 5 %( 31) mothers at Debre-sina Hospital for pre-test and re-edited some questions.
- We have trained five midwifery professionals.
- We verified the completeness of the collected data.
- We entered the collected data into epi-data version 4.6 and then transferred it to SPSS.
- We entered the data for the missed variable check in SPSS and re-coded and transformed the variable into a different variable. Respected, because as we have mentioned above, our quality of paper was better.

**4. Do you think the problem is solved after recommend this result for the concerning bodies, due to study design and data analysis that you were exercised.**

Answer

- Of course, the magnitude of MSAF found in our study has increased from before; we have identified the factors that caused MSAF to increase, so we believe that they will pay attention to the problem, future researchers will study with a better study design, and the problem will be solved.

5. What is the implication of the study in the study area in particular and worldwide in general.

Answer

This is a beautiful question.

- This study will be used as a resource for the health professionals in the North Shoa; that is, it will inform them of the amount of MSAF in the zone and its factors, and they will reduce the morbidity and mortality caused by this. It will also help them plan and implement their work.
- This study can be used to study other studies, prepare projects, and analyze related health data, both locally and internationally. Also, policymakers can use it as a basis.

**6. How can co relate for the recommendation of "fetal tachycardia(AOR=6.2; 95% CI; 2.41-16.3) and Bradycardia (AOR=3.1; 95% CI;1.93-5.28) were significant association with meconium stained amniotic fluid".**

Answer

- As we know, if there is fetal stress, it means an increase or decrease in the fetal heart rate. Therefore, if the FHR is higher than normal, the baby is very stressed in the mother's womb, and that is why there is meconium. Likewise, if the FHR is low, we know that there is meconium. In conclusion, whether the FHR is increased or decreased, we know that the baby is stressed and there is meconium.

**7. How can interpret and possible to recommend by cross sectional study of the following statements: “Women had mid-upper arm circumference value was less than 22.9cm(AOR=1.9; 95% CI;1.18-3.20)".**

Answer

Thanks for this important question.

- Although the cross-sectional study did not provide strong evidence as a follow-up study, we indicated an association between MSAF and maternal nutritional status. That is, we observed in this study that mothers are at risk for MSAF if their nutritional status is measured by MUAC <22.9cm low status, whereas 23-27.5cm is normal nutritional status and >27.6cm is normal above nutritional status. Therefore, although this study is explicatory, we know that low nutritional status is the cause of MSAF. In the future, we recommend that this study be studied with a better study design, and this study can be used as a basis.

**Response to Reviewer # 2**

**1. Background: Line number 22 &23: Says “In Ethiopia meconium-stained amniotic fluid is under investigated specifically in North Shoa Zone Amhara region Ethiopia”. Do you think that researches for each problem should be done in each zone of the region to say “well investigated”? Why the research findings in other zones of the region like North Gondar, East Gojjam South Wollo…cannot be applicable for North Shoa?**

Answer

- In fact, we have accepted this opinion and tried to correct it, but dear, what we wanted to say in this article was not as you understood; the studies that were studied on other zones in the Amhara region described North Shoa, but because they studied most of the studies only on a single hospital, this does not give us enough and accurate information about MSAF. Therefore, we believe that all ten hospitals in North Shoa should be studied.

**2. Methods: Line 27 &28 : “Two stage cluster sampling was used to recruit study participants”. Is cluster sampling technique appropriate for your research? Why you prefer it over systematic random sampling technique? Would you mind attaching the schematic presentation of the sampling procedure? Why you use a design effect of 2?**

Answer

Once again, we are greatly appreciative, as we believe that this question is the backbone of our research publication. It is known that there are three types of cluster sampling: one-stage clusters, two-stage clusters, and multi-stage clusters. As a result, we used a two-stage cluster for our study because

- The communities living in North Shoa are homogenous.
- There are 10 hospitals in different places in the zone, which means that the people who live under each hospital are considered to be a cluster, which means we have 10 clusters in the zone. For this reason, we selected five of the 10 hospitals in the zone by simple random sampling and allocated them proportionally to each of them.
- The participants of our study are immediate postpartum women, so we used systematic random sampling based on the delivery registration book to select the study participants from each hospital. So ***we have two stages***: we ***used two different sampling*** ***techniques*** (systematic and simple random sampling) at the same time. If these things exist, ***sampling variation is inevitable***, so we are forced to use design effect 2.

**NOTE:** For more information, please see our schematic presentation of the sampling procedure.

**Sampling procedure**

**APH**

**MGH**

**198**

**MGH**

**83**

**Annually North shoa zone hospitals give delivery service for =12914**

**DBCSH**

**DSPH**

**SRPH**

**ATPH**

**MPH**

**MGH**

**MWPH**

**EGH**

**DPH**

DBCSH

**591**

**ATPH**

**230**

**SRPH**

**224**

**EGH**

**174**

**DBCSH**

**284**

**ATPH**

**96**

**SRPH**

**93**

**EGH**

**72**

**628**

Figure 1 schematic presentation of sampling technique on magnitude of Meconium stained amniotic fluid and associated factors among women who gave birth in North Shoa zone Amhara region Ethiopia October 2022.

**3. Conclusion: Line 38: “The magnitude of meconium-stained amniotic fluid, which was high”. What is your ground/reference frame to say “high”? What is “low” to you? Your recommendation is not in line with your finding: Did your research finding showed a gap on “intrapartum service” and “a delay on detecting meconium-stained amniotic fluid “ in the study area?**

Answer

Here, we have accepted your comments.

- The amount of MSAF that we said was high was compared to what was studied in Ethiopia in the past, but now we have corrected it.
- We did not see a problem in detecting MSAF during intrapartum, but we have called those with low nutritional status prone to MSAF, so they should measure MUAC during ANC follow-up.
- We recommend to other associated obstetrical-related complications professionals that they pay more attention to MSAF during childbirth than usual, which we believe is in line with the results. We also recommend that they do prospective cohort studies.

**4. Introduction: line number 45-53: More than 9 lines/ two paragraphs taken from one source without paraphrasing it which is not appropriate writing.**

Answer

- We have fully accepted your feedback and rewrite the paragraph.

**5. Methods and materials: line 80: Better to incorporate how many Hospitals are there in your study setting and number of deliveries in each Hospital monthly.**

Answer

- We have fully accepted your comments and have listed all the hospitals and stated how much delivery care each hospital provides every two months.

**6. Line 99: I am not comfortable with design effect and cluster sampling technique. I need a strong justification with schematic presentation of sampling procedure.**

Answer

We decided that we should use design effect when writing a proposal to explore this study. The reason is

- There is no doubt that there is sampling variation here, so we had to increase the sample size by using design effect.
- We have employed two different sampling techniques together. The main thing is that since we have used a large sample size, it will not have a negative impact on our study.

**NOTE**: - the schematic presentation provided in response for question number two can support the concern on sampling techniques and design effect.

**7. Line 125: what were the adjustments and modifications made to the tool after your pre-test?**

Answer

- When we did the pre-taste, we only revised a few questionnaires because most of our questionnaires were good.

**8. Lines 137-145: All your operational definitions citation**

Answer

- We have received your comments and made citations for all our operational definitions.

**9. Result: Table 2: Have you ever drinking Alcohol? Yes (69), No (541) and Have you drinking Alcohol currently Yes? (148), No ( 457). A paradox result,**

Answer

- You are right; the number of mothers who drank alcohol before pregnancy is 71 higher than that of those who drank it during pregnancy. But what we can assure you is that this shows the actual reality on the ground, because pregnant mothers, especially in rural areas, like to drink alcohol, which is a traditional drink, saying that it makes us safe, makes us joyful, and is good during labor. Indeed, many of them do not say that they drink because it is medically prohibited for pregnant women, but we believe that it is more than 148. However, as you have seen it, so that it does not seem like a paradox to the readers, we thought to leave it in the document as far as the text of the table raw that says "ever drinking alcohol and ever chewing khat."

10. Can we measure behavioural factors with simple yes/no questionnaire?

Answer

- In fact, this is an important opinion. As you know, we can measure substance measurement and alcohol using the ASSIT and/or AUDIT tools, but this does not work for pregnant women, so we modified question number two of the ASSIT tool and prepared a yes/no question. So far, these substance users are pregnant women. We believe they should be studied because the studies to date have not at least studied them using Yes/No. However, we accept your comments, and we intend to leave them or explain them on our weak side.

**11. Table: Can it add a value to your result using “gravidity and parity” at the same time as different factors? Look at your result again “Gravidity 2-4=305 and parity 2-4=358, how it could be? Is parity greater than gravidity?**

Answer

- When we did the pre-test, we only revised a few questionnaires because most of our questionnaires were good. In fact, we are very sorry for the numerical error that we put in the article we wrote here. But now we believe that you have given your opinion (***Can it add a value to your result using “gravidity and parity”***) and we leave it. However, this is our correct table (show table 1 below).

Table 1 reference for our previous error

| Variables | Category | frequency | Percent (%) |
| --- | --- | --- | --- |
| Gravidity | 1  2-4  ≥5 | 230  358  22 | 37.7  58.7  3.6 |
| Parity  (out of 610/30 women do not have parity) | 1  2-4  ≥5 | 223  336  21 | 38.4  57.8  3.8 |

**12. Discussion: The literatures compared to your result should be written from local to global unlike that of literature writing style.**

Answer

- We have fully accepted this comment and rewrite the discussion part from global to local.

***With Many Thanks***

***We are looking forward seeing your next responses***

***Authors,***
