# Supplementary material for: Magnitude of meconium stained amniotic fluid and associated factors among women who gave birth in North Shoa Zone hospitals, Amhara Region, Ethiopia 2022
Source: PLoS One. 2024 Feb 14;19(2):e0297654. doi: 10.1371/journal.pone.0297654 (PMC10866510; doi:10.1371/journal.pone.0297654)
Supplement: Attachment — Submitted filename: commnets to authors.docx [file pone.0297654.s008.docx]

Dear editor thank you so much for your invitation to review this article. The whole work of this study sounds scientific. However, in my point of view, this article doesn’t merit publication in its present status. So I suggest the authors to amend it before online publication. Please, find my comments and suggestions hereunder.

Title: Authors would study an obstetric condition that causes meconium-stained amniotic fluid because it is a sign of underlying obstetric conditions. Fetal distress due to obstructed labour and prolonged labour or other labour complications are the causes of MSAF.

Abstract: line 32 & 33 incomplete sentence.

Introduction

1. This section would mention the previous interventions undertaken (if any) to lessen the problem or any directed plan against it.
2. “Based on this study, health practitioners can identify high-risk labouring women and newborns early and take 75 prompt action to improve their outcomes with an understanding of the magnitude and associated factors of 76 MSAF.” Is your study found any MSAF diagnostic modality? What new antecedents of MSAF did your study added to the field? I think all professionals in the field know that anaemia, malnutrition, obstructed and prolonged labour, PROM, bradycardia, and tachycardia predispose to MSAF.

Materials and Methods:

1. Why did you exclude breech deliveries? Authors missed most important cases because such cases are risky for labour complications which can lead to MSAF. Congenital malformations are also risk factors for labour complications.
2. The authors mentioned that they used a two-stage cluster sampling. Why cluster sampling? What are the assumptions of cluster? Note that this sampling technique is exceptionally vulnerable for sampling error compared to other probability sampling techniques?
3. You mentioned that you got the delivery registration book (as a sampling framework) but still you have applied a systematic random sampling technique that less representative than a simple random sampling.
4. Line 125 to 127 “To reduce translation errors, the data collector used the Amharic version of the questionnaire to collect data from the delivery logbook or chart” Factually, medical and obstetrics histories and reports are documented in English. So how did you collect it in Amharic language and what is its importance? The Amharic language might only be used to interview the mothers.
5. The data collection techniques where not clearly mentioned. It seems the authors approached the postpartum women. If so, in which interval postpartum period? How did you approached and interviewed very exhausted postpartum mother? You also interviewed women who underwent C/S surgery, so how did you interview those unresponsive women due to the anaesthesia effect? These should be briefed.
6. What other measurements were taken to maintain data quality? Didn’t the data collectors and supervisors trained?
7. Authors mentioned that they checked multicolliniarity but they didn’t mention the techniques they used.
8. How did you measure the degrees of MSAF? Note that it is a subjective observation of the colour and appearances of the AF. So would think the documented diagnosis, observed by different individuals, were reliable?
9. You didn’t cite sources for your term definitions. Almost all are incorrect citations.
10. Is it “protocol” or “reference number”?
11. The ethical clearance and consent procedure was not coherently written.
12. Line 154 no “Informed consent was obtained for Section 1 (socio-demographic and behavioral factors) from the…” Was your informed consent applied for a certain part of questions? Why?
13. Authors mentioned that they took a verbal consent from participants. So you would elaborate the procedures you have followed to do that.

Results

1. There some cells with observed value less than 0.5% in table.
2. The subsection “Behavioral factors” would be replaced by “Substance use”. And also you would study an important variable ‘smoking’ which is risky for fetal distress and labour complications. Note that smoking causes compromises fetal oxygenation. It also causes hypertrophy due to prolonged placental tissue hypoxia which can lead to placenta previa; in turn leads to labour obstruction. You missed it.
3. There were missed observations for alcohol drinking. Check Table 2
4. Some of the variables in table 4 are put in questions forms. Please correct it.
5. You would include the proportion for each variable the cross tab (table 5).

Discussion

1. The first elaboration you gave for the higher proportion of MSAF in the area is not plausible. Let you check it.
2. “Therefore, it was confirmed that this study showed a more truthful picture than studies done in different areas of Ethiopia for all the reasons mentioned above” Please do not use such type expressions.
3. Most of the authors’ interpretations and elaborations are not plausible. Please, check this sections

As it was a clinical study, authors

Acknowledgment

1. Authors would acknowledge the study participants, data collectors, and supervisors who played unwavering efforts in the study’s accomplishment.
2. I think it is not necessary to acknowledge a journal for their peer review service.

Generally, the manuscript is full of grammatical, sentence, and punctuation errors. I recommend the authors correct the mentioned issues. Although the previous reviewers mentioned the same points, you didn’t amend it.
