# Supplementary material for: Magnitude of meconium stained amniotic fluid and associated factors among women who gave birth in North Shoa Zone hospitals, Amhara Region, Ethiopia 2022
Source: PLoS One. 2024 Feb 14;19(2):e0297654. doi: 10.1371/journal.pone.0297654 (PMC10866510; doi:10.1371/journal.pone.0297654)
Supplement: Attachment — Submitted filename: Response to reviewers.docx [file pone.0297654.s010.docx]

**Letter of response**

**To all honourable reviewers and editors**

Dear Reviewers and Editors, We are very grateful for your relevant comments, questions and suggestions. Following this, we have provided our responses point by point as presented below.

**Authors Responses to Reviewer #4:**

Dear editor thank you\ so much for your invitation to review this article. The whole work of this study sounds scientific. However, in my point of view, this article doesn’t merit publication in its present status. So I suggest the authors to amend it before online publication. Please, find my comments and suggestions hereunder.

Title: Authors would study an obstetric condition that causes meconium-stained amniotic fluid because it is a sign of underlying obstetric conditions. Fetal distress due to obstructed labour and prolonged labour or other labour complications are the causes of MSAF.

Abstract: line 32 & 33 incomplete sentence.

**Authors Responses**

- In fact, we have accepted this opinion and tried to correct the whole part our abstract write-up

Introduction

1. This section would mention the previous interventions undertaken (if any) to lessen the problem or any directed plan against it.

**Authors Responses**

- Once again, we are greatly appreciative, as you suggested we are try to mention that is not put interventions across previous related to studies done on MSAF.

2. “Based on this study, health practitioners can identify high-risk labouring women and newborns early and take 75 prompt action to improve their outcomes with an understanding of the magnitude and associated factors of 76 MSAF.” Is your study found any MSAF diagnostic modality? What new antecedents of MSAF did your study added to the field? I think all professionals in the field know that anaemia, malnutrition, obstructed and prolonged labour, PROM, bradycardia, and tachycardia predispose to MSAF.

**Authors Responses**

- This is an interesting question. Your suggestion is fully accepted. As far as we have not studied diagnostic modality of MSAF, but it can increase the alertness of health practitioners through the understanding of the magnitude and associated factors of MSAF.

Materials and Methods:

1. Why did you exclude breech deliveries? Authors missed most important cases because such cases are risky for labour complications which can lead to MSAF. Congenital malformations are also risk factors for labour complications.

**Authors Responses**

- Of course we excluded breech presentation and congenital malformations because of many evidences in the web showing breech presentation and congenital malformations as normal fatal condition in the presence of MSAF during labour and delivery. In the presence of breech presentation and congenital malformations, the outcome of baby is normal, so we have excluded breech presentation and congenital malformations.

2. The authors mentioned that they used a two-stage cluster sampling. Why cluster sampling? What are the assumptions of cluster? Note that this sampling technique is exceptionally vulnerable for sampling error compared to other probability sampling techniques?

**Authors Responses**

Once again, we greatly appreciate your comments, and consider others reviewer idea and amend it with multistage sampling procedure, since it does not affect the result of the study.

3. You mentioned that you got the delivery registration book (as a sampling framework) but still you have applied a systematic random sampling technique that less representative than a simple random sampling.

**Authors Responses**

Dear reviewers thanks for your glad suggestion

- As we mentioned in our manuscript, we applied both simple random and systematic random sampling methods because the participants of our study are immediate postpartum women, so we used systematic random sampling based on the delivery registration book to select the study participants from delivery registration book and we used simple random sampling to select 5 hospitals from 10 hospitals. As you said a systematic random sampling technique that less representative than a simple random sampling. Due to this reason we are forced to use design effect to reduce sampling error and it can be more representative.

4. Line 125 to 127 “To reduce translation errors, the data collector used the Amharic version of the questionnaire to collect data from the delivery logbook or chart” Factually, medical and obstetrics histories and reports are documented in English. So how did you collect it in Amharic language and what is its importance? The Amharic language might only be used to interview the mothers.

**Authors Responses**

Respected, you are right and we accepted

But, we have two type of questioners that means the first 15 questions were are Amharic version and we interview the mothers face to face. The remaining of all question were English version and we used for maternal document review.

5. The data collection techniques where not clearly mentioned. It seems the authors approached the postpartum women. If so, in which interval postpartum period? How did you approached and interviewed very exhausted postpartum mother? You also interviewed women who underwent C/S surgery, so how did you interview those unresponsive women due to the anaesthesia effect? These should be briefed.

**Authors Responses**

Respected, we accepted it

- As we mentioned, we interviewed postpartum mother for the first 15 questionaries’ but, we asked the women after 6 hr of delivery for women who underwent C/S surgery and very exhausted postpartum mother.

6. What other measurements were taken to maintain data quality? Didn’t the data collectors and supervisors trained?

**Authors Responses**

Thank you for your nice inquiry

- Five midwifery professionals trained and employed for the data collection. The questionnaire was distributed to 31 women at Debre-Sinai Hospital (5% of the sample size) before the real data collection began. The main investigator reviewed the data to ensure its completeness and consistency, and we have rectified few sentences on the tool.

7. Authors mentioned that they checked multicolliniarity but they didn’t mention the techniques they used.

**Authors Responses**

- Really we accepted and amended it accordingly

8. How did you measure the degrees of MSAF? Note that it is a subjective observation of the colour and appearances of the AF. So would think the documented diagnosis, observed by different individuals, were reliable?

**Authors Responses**

- Respected, you are right, but in our study the degrees of MSAF was taken from the chart which was confirmed by the physicians or any other legitimate health professional during the data collection period. In fact, there exists a variation among physicians during diagnosis.

9. You didn’t cite sources for your term definitions. Almost all are incorrect citations.

**Authors Responses**

- The respected reviewer amended it accordingly, and we have four term definitions and cited our references (articles that we adopted). But we asked apologise to you for our previous mistake—that we didn’t update our reference software. Now we have solved these problems and updated the whole reference.

10. Is it “protocol” or “reference number”?

**Authors Responses**

- Dear reviewer we have used mendeley reference software but we accepted and amended your suggestion.

11The ethical clearance and consent procedure was not coherently written.

**Authors Responses**

- Dear reviewer we accepted and amended it accordingly.

10. Line 154 no “Informed consent was obtained for Section 1 (socio-demographic and behavioral factors) from the…” Was your informed consent applied for a certain part of questions? Why?

**Authors Responses**

- We accepted and amended it accordingly. We would receive the informed consent for all type of questioners.

11. Authors mentioned that they took a verbal consent from participants. So you would elaborate the procedures you have followed to do that.

**Authors Responses**

Respected reviewer

- First we have been received ethical approval from the institutional review board of the Asrat Weldeyes Health Science Campus. Then, the purpose of the study was explained to the study participants; confidentiality was ensured; and for all study participants, verbal consent was obtained prior to data collection. At all levels, officials were contacted, and permission has been secured.

Results

1. There some cells with observed value less than 0.5% in table.

**Authors Responses**

- Respected, you are right, but here is in our frequency table merged and omitted that cell less than 0.5% that is fulfil the assumption.

2. The subsection “Behavioral factors” would be replaced by “Substance use”. And also you would study an important variable ‘smoking’ which is risky for fetal distress and labour complications. Note that smoking causes compromises fetal oxygenation. It also causes hypertrophy due to prolonged placental tissue hypoxia which can lead to placenta previa; in turn leads to labour obstruction. You missed it.

**Authors Responses**

- We accepted and merged these behavioural factors with socio-demographic factors. Considering your suggestion, smoking is an important variable, but we considered it to have more social desirability bias than other substance use in rural parts of Ethiopia. However, now we feel your comment because we missed it.

3. There were missed observations for alcohol drinking. Check Table 2

**Authors Responses**

- We accepted and amended our writing error accordingly

4. Some of the variables in table 4 are put in questions forms. Please correct it.

**Authors Responses**

- We accepted and amended our writing error accordingly

5. You would include the proportion for each variable the cross tab (table 5).

**Authors Responses**

- We accepted and amended it accordingly.

Discussion

1. The first elaboration you gave for the higher proportion of MSAF in the area is not plausible. Let you check it.

**Authors Responses**

- We accepted and amended our writing error accordingly

2. “Therefore, it was confirmed that this study showed a more truthful picture than studies done in different areas of Ethiopia for all the reasons mentioned above” Please do not use such type expressions.

**Authors Responses**

- We accepted and amended it accordingly.

3. Most of the authors’ interpretations and elaborations are not plausible. Please, check this sections

**Authors Responses**

- We accepted and amended it accordingly.

Acknowledgment

1. Authors would acknowledge the study participants, data collectors, and supervisors who played unwavering efforts in the study’s accomplishment.
2. I think it is not necessary to acknowledge a journal for their peer review service.

Generally, the manuscript is full of grammatical, sentence, and punctuation errors. I recommend the authors correct the mentioned issues. Although the previous reviewers mentioned the same points, you didn’t amend it.

**Authors Responses**

- We accepted and amended it accordingly.

**Response to Comments to the Author**

1. If the authors have adequately addressed your comments raised in a previous round of review and you feel that this manuscript is now acceptable for publication, you may indicate that here to bypass the “Comments to the Author” section, enter your conflict of interest statement in the “Confidential to Editor” section, and submit your "Accept" recommendation.

Reviewer #3: (No Response)

Reviewer #4: (No Response)

**Authors Responses**

- Respected editor we accepted and amended your comments accordingly but we are very committed to amend for next comment

2. Is the manuscript technically sound, and do the data support the conclusions?

The manuscript must describe a technically sound piece of scientific research with data that supports the conclusions. Experiments must have been conducted rigorously, with appropriate controls, replication, and sample sizes. The conclusions must be drawn appropriately based on the data presented.

Reviewer #3: Yes

Reviewer #4: Yes

3. Has the statistical analysis been performed appropriately and rigorously?

Reviewer #3: Yes

Reviewer #4: Yes

4. Have the authors made all data underlying the findings in their manuscript fully available?

The [PLOS Data policy](http://www.plosone.org/static/policies.action#sharing) requires authors to make all data underlying the findings described in their manuscript fully available without restriction, with rare exception (please refer to the Data Availability Statement in the manuscript PDF file). The data should be provided as part of the manuscript or its supporting information, or deposited to a public repository. For example, in addition to summary statistics, the data points behind means, medians and variance measures should be available. If there are restrictions on publicly sharing data—e.g. participant privacy or use of data from a third party—those must be specified.

Reviewer #3: Yes

Reviewer #4: Yes

5. Is the manuscript presented in an intelligible fashion and written in standard English?

PLOS ONE does not copyedit accepted manuscripts, so the language in submitted articles must be clear, correct, and unambiguous. Any typographical or grammatical errors should be corrected at revision, so please note any specific errors here.

Reviewer #3: Yes

Reviewer #4: No

**Authors Responses**

- We accepted it, have tried to revise language editing through the document, and it will further be edited in the process of writing it up for publication.

***With Many Thanks***

***We are looking forward seeing your next responses***

***Authors,***
